# Efficacy and Safety of Tripterygium Glycoside in the Treatment of Diabetic Nephropathy: A Systematic Review and Meta-Analysis Based on the Duration of Medication

**DOI:** 10.3389/fendo.2021.656621

**Published:** 2021-04-20

**Authors:** Yizhen Li, Runpei Miao, Yixing Liu, Jiawei Zhang, Zhili Dou, Lei Zhao, Yunan Zhang, Zhe Huang, Ye Xia, Dongran Han

**Affiliations:** ^1^ School of Life and Science, Beijing University of Chinese Medicine, Beijing, China; ^2^ School of Traditional Chinese Medicine, Beijing University of Chinese Medicine, Beijing, China; ^3^ School of Management, Beijing University of Chinese Medicine, Beijing, China

**Keywords:** tripterygium glycosides, meta-analysis, systematic review, diabetic nephropathy, medication safety

## Abstract

**Aim:**

The aim of this study was to assess the clinical efficacy and safety of Tripterygium-derived glycosides (TG) after 3-month and 6-month of treatments of diabetic nephropathy (DN) and to resolve the conflict between medicine guidance and clinical practice for TG application.

**Methods:**

We conducted a systematic review and meta-analysis of randomized controlled trials involving TG application in treating DN. We extensively searched PubMed, Cochrane Library, CNKI, VIP, Wan-Fang, CBM, Chinese Clinical Trial Registry, and WHO International Clinical Trial Registration Platform till November 2020, along with grey literature for diabetes and all other relevant publications to gather eligible studies. Based on the preset inclusion and exclusion criteria, document screening, quality assessment of methodology, and data extraction was conducted by two researchers independently. The methodological quality was assessed by the Cochrane risk test from the Cochrane Handbook 5.2, and then analyses were performed by Review Manager 5.3 (Rev Man 5.3). The quality of output evidence was classified by GRADE.

**Results:**

Thirty-one eligible studies (2764 patients) were included for this meta-analysis. Our study results showed a comparable significant decrease in the 24 h-UTP and blood creatinine levels in DN patients from both 3-month and 6-month TG treatment groups, compared with the routine symptomatic treatment alone. To the contrary of the findings from the included studies, our results showed that the occurrence of serious adverse reaction events was significantly higher in the TG treated group with 6 months of treatment duration compared to that of 3 months of the treatment course. However, the total AR ratio was slightly varied while increasing the percent of severe adverse events. GRADE assessment indicated that the quality of evidence investigating TG-induced adverse reactions was moderate and that for 24 h-UTP and blood creatinine indicators were considerably low.

**Conclusion:**

Combinatorial treatment regimen including TG can significantly decrease the pathological indicators for DN progression, while it can also simultaneously predispose the patient to a higher risk for developing severe adverse events, as the medicine guidance indicates. Notably, even in 3-month of course duration smaller percent of severe adverse events can get to a fatal high percent and is likely to increase proportionally as the TG treatment continues. This suggests that TG-mediated DN treatment duration should be optimized to even less than 3 continuous months to avoid adverse event onset-associated further medical complications in DN patients. In clinical practice, serious attention should be paid to these severe side-effects even in a course normally considered safe, and importantly more high-quality studies are urgently warranted to obtain detailed insights into the balance between the efficacy and safety profiles of TG application in treating DN.

## Introduction

Diabetic nephropathy (DN) is characterized by degeneration of the renal microvasculature leading to leakage of proteins like albumin into the urine (commonly known as proteinuria), perturbed glomerular filtration, increased fluid retention, and high arterial blood pressure (1). DN frequently occurs in patients with diabetes mellitus, and its symptoms indicate chronic end-stage renal failure. DN is also considered as the leading cause of death in patients with chronic renal failure, with a prevalence rate of 4.8% (1, 2). Current therapeutic regimens include angiotensin II receptor blocker (ARB) and inhibitors for angiotensin-converting enzyme (ACE) to reduce the high blood pressure-associated renal complications and progression to DN (3, 4). Although some studies claim that ARBs are effective in treating proteinuria or albuminuria than ACE inhibitors, however, there are also contradictory results showing both of them have very similar efficacy in reducing proteinuria symptoms in primary hypertensive patients (5, 6). Despite the routine clinical applications of these drugs for slowing down DN progression, it has been challenging to reduce proteinuria completely in both diabetic- and non-diabetic patients with DN, particularly in cases of severe proteinuria. Furthermore, ARB/ACE inhibitors can induce fatal side-effects in patients with advanced stages of chronic renal insufficiency and having a serum creatinine level greater than 3mg/dL (7).

Hyper-activation of inflammatory responses has been frequently observed in patients with diabetes-related renal dysfunctions or chronic renal insufficiency (8), which further complicates the pathogenesis of DN (9–11). Recent investigations have greatly explored effective strategies for inhibition of renal infiltration of activated immune cells, cytokine storm, inflammatory responses, apoptosis, and podocyte injury as well to prevent DN progression (9, 12, 13). Notably, recently developed reno-protective drugs, such as sodium-glucose cotransporter-2 (SGLT-2) inhibitors, also have shown promising anti-inflammatory and anti-oxidative stress effects in the treatment of DN (14).

Tripterygium glycosides (TG) is an active compound found in the root extracts of *Tripterygium wilfordii* (TW). TG has been an essential component of traditional Chinese medicine for the treatment of glomerulonephritis and as a powerful immunosuppressive agent during kidney transplantation. TG has been increasingly utilized in the treatment of DN mainly due to its anti-inflammatory functions as well as its superb ability to prevent oxidation-induced membrane disruption in the glomerulus, thereby preventing DN progression and proteinuria (15–17). Moreover, combined therapy, including TG and ARB/ACE inhibitors, have been clinically applied as a potential therapeutic regimen against DN symptoms (18, 19).

In spite of multimodal pharmaceutical benefits of TG in treating several chronic life-threatening diseases, including DN and rheumatoid arthritis, TG-induced adverse events (AEs) have been mostly found to be systemic, organ-specific depending on the drug dose and duration of medication course (20). According to the drug overdose-related guidelines for TG tablets (Hunan Xieli Pharmaceutical Co. Ltd. National Medicine Standard Z43020138), long-term administration of TG may impact a number of physiological functions, e.g., digestion, blood pressure, renal and cardiovascular dysfunctions as TG-induced AEs, thus recommending a safe dose and course duration for 3 months. However, several other groups have shown no significant differences in safety profiles between 3 months and 6 months of treatment duration (21). Furthermore, recent studies investigating the impacts of different doses of TG in treating DN has revealed that 60 mg/day of TG is more effective than 30 mg/day for a period of 6 months with the same safety profile (22), and also TG is more efficient than valsartan in reducing the proteinuria level (23). The study also reports that there was no significant difference in AE occurrences between the placebo-treated and low-dose TG treated groups; however, in the double-dose group, only one patient exhibited an elevated level of alanine aminotransferase but was less than 2-fold compared to the baseline level, which was immediately normalized after symptomatic treatment.

Controversies exist between systematic reviews that focus on the clinical efficacy and adverse reactions of TG in the treatment of DN. Some researchers believe that TG can improve some clinical indicators of patients with DN, such as 24 h-UTP and serum creatinine level (24–26), while others reached negative conclusions that TG can significantly induce adverse reactions in patients with DN (27, 28), and even questioned whether TG can be used to reduce the serum creatinine level of patients (28).

Thus, in this meta-analysis and systematic literature review regarding the safety and efficacy of TG administration in DN treatment and its relation to the duration of medication course, we have investigated the immediate necessities for “how” and “when” to balance between the standard medicine usage guidelines and empirical therapies for TG. This study will be highly beneficial to lay the theoretical foundations and practical clinical applications of TG to cure DN.

## Materials and Methods

### Methods

This meta-analysis was conducted following the guidelines of the Preferred Reporting Items for Systematic Reviews and Meta-Analysis (PRISMA). Prior ethics approval and consent of the participants were not required for this study since it involved only previously published RCT studies.

### Literature Search Strategy

We performed a systematic search on PubMed, Cochrane Library, and WHO International Clinical Trial Registration Platform (ICTRP) for English language publications and China National Knowledge Internet (CNKI), China Science & Technology Journal Database (VIP), Wan-Fang digital periodical full-text database, Chinese Biomedical Literature Database (CBM) and Chinese Clinical Trial Registry (ChiCTR) for Chinese publications from database inception to November 05, 2020 based on the defined inclusion and exclusion criteria. The predefined English terms used for the search included “diabetic nephropathy” or “diabetic kidney disease” and “tripterygium glycosides” and terms related to randomized controlled trials (RCTs). In addition, we searched both manually and electronically for potentially eligible abstracts of newspapers, grey literature in the field of diabetes, along with any associated e-magazine references in order to prevent from missing any relevant studies. All the literature was published before November 2020. The detailed search strategy is provided in the supporting information.

### Selection Criteria

Relevant studies were carefully screened by abstracts and titles, and then the eligibility criteria were applied based on PICOS as follows: (1) Patients: patients diagnosed with DN. The diagnostic criteria of DN was in accordance with 2007 National Kidney Foundation Kidney of Disease Outcomes Quality Initiative (NKF-K/DOQI): That is, in most patients with diabetes, the kidney damage should be considered as caused by diabetes if any of the following conditions occurs: massive proteinuria, diabetic retinopathy with microalbuminuria, microalbuminuria occurs in type 1 diabetes with the course of diabetes lasting for more than 10 years. (2) Intervention: TG combined with basic treatment applied, and the duration of TG treatment lasted for 3 or 6 months. (3) Comparison: TG combined with the basic treatment comparing with the basic treatment. (4) Outcomes: The efficacy of primary outcome was assessed by the changes in 24 h-urine total protein (24 h-UTP) and blood creatinine levels. (5) Study design: randomized controlled trials (RCTs) that applied TG in conventional treatment for DN for 3 months or 6 months of course duration, regardless of English or Chinese language, year of publication or country of publication.

Records retrieved from electronic searches were imported into reference management software (EndNote X7, Thomson Reuters, New York, NY, USA). After removing duplicate records, two reviewers independently screened the titles and abstracts of the remaining reports and subsequently investigated potentially eligible studies in full text. Inclusion and exclusion criteria are presented in **Table S1**. Differences in opinion between the two independent reviewers at any stage of the study processes were resolved by their mutual consensus or were further arbitrated by a third reviewer to reach a consensus.

### Data Extraction and Risk-of-Bias Assessment

Two researchers extracted data independently, any discrepancies were discussed and resolved after consulted with a senior researcher. For each eligible trial, data on study characteristics, participants’ baseline characteristics, key efficacy, and safety outcomes were extracted.

The risk of bias for the primary outcome was assessed by the respective tool developed by the Cochrane Collaboration 5.2. In this assessment, the following domains were considered: random sequence generation, allocation concealment, blinding of participants and personnel, blinding of outcome assessment, incomplete outcome data, selective reporting, and other biases. The risk of bias for every domain was rated as high, unclear or low independently. Key domains included random sequence generation, allocation concealment, and incomplete outcome data. Publication bias was tested by funnel plot symmetry when at least 10 studies were available per meta-analysis.

### Data Synthesis and Statistical Analysis

The extracted data were analyzed separately for 3-month treatment or 6-month treatment durations. We used the relative risk (RR) with 95% confidence intervals (CI) for dichotomous data, mean difference (MD) with 95% CI for continuous outcomes, heterogeneity quantified as high with I^2^ values >50% and *p*<0.1. If substantial heterogeneity existed, a random-effects model was used to pool measures; otherwise, a fixed-effects model was used. Statistical analyses were performed using RevMan 5.3 (Nordic Cochrane Center, Copenhagen, Denmark, 2014).

## Results

### Search Results and Study Characteristics

**Figure 1:**
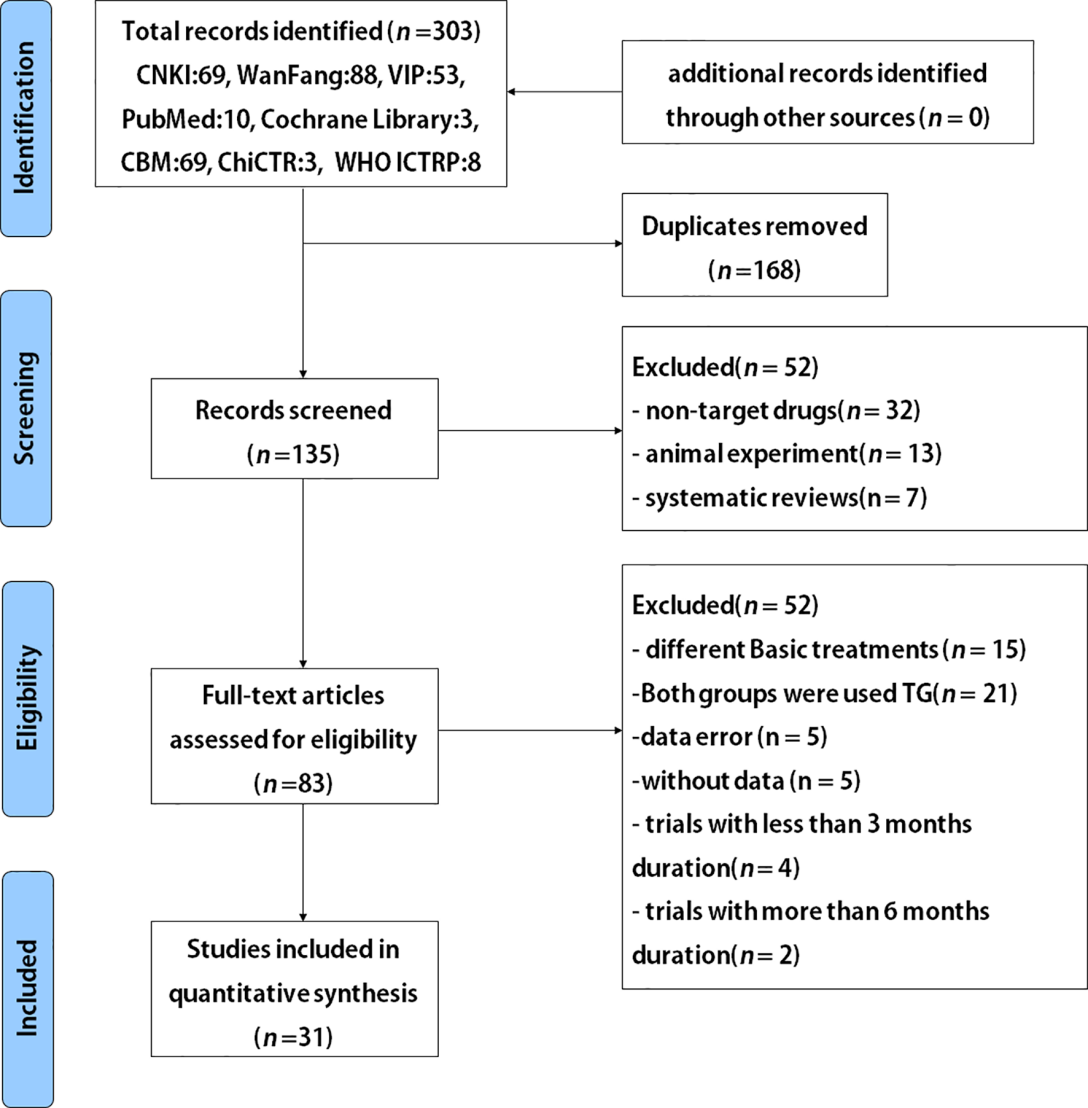
Flowchart for randomized controlled trials (RCTs) to evaluate the use of TG after 3 and 6 months of treatment durations in patients with DN.

The study selection process is depicted in **Figure 1**. A total of 31 studies with 2764 participants were included after careful screening and evaluation for the systematic review (22, 29–58) (**Table 1**). All the studies were published in full-text, and participants’ characteristics are shown in **Table 1**. The mean ages across the studies were ranged from 34.6 to 69.5 years, and 46.9% (1295) of the overall participants were women. Courses of treatment included were 3 months and 6 months. In all included trials TG combined with routine symptomatic treatments were compared with the symptomatic treatments alone. The symptomatic treatment targets included control of blood pressure and blood sugar, reduction of urinary protein content and blood creatinine level, etc. All studies exhibited that the baseline results for the TG-treated group and the corresponding control group were comparable.

**Table 1:** Characteristics of studies and participants included in systematic review and meta-analysis.

Study, year [reference]	Number of participants	Dose of TG	Study duration, months	Outcomes of 24h-UTP and blood creatinine	Adverse event report
Li et al., 2012 (29)	128	60 mg/d	3	24h-UTP	Not reported
Lu et al. 2020 (30)	100	60 mg/d	3	Not reported	YES
Wang et al. 2013 (31)	64	-	3	24h-UTP, blood creatinine	YES
Gai et al. 2020 (32)	104	60 mg/d	3	24h-UTP, blood creatinine	No adverse event
Zhu et al. 2018 (33)	180	1mg/(kg·d)	3	24h-UTP, blood creatinine	YES
Wang et al., 2017 (34)	60	60 mg/d	3	Not reported	YES
Sun et al., 2019 (35)	100	1mg/(kg·d)	3	blood creatinine	YES
Yan et al., 2017 (36)	92	60 mg/d	3	Not reported	No adverse event
Liu et al., 2015 (37)	60	1mg/(kg·d)	3	blood creatinine	YES
Wang et al., 2018 (38)	80	60 mg/d	3	24h-UTP	No adverse event
Liu et al., 2015 (39)	40	1mg/(kg·d)	3	24h-UTP	YES
Zhang et al., 2015 (40)	40	1mg/(kg·d)	3	24h-UTP, blood creatinine	No adverse event
Shen et al., 2011 (41)	90	1mg/(kg·d)	3	24h-UTP, blood creatinine	YES
Shi et al., 2018 (42)	81	60 mg/d	3	24h-UTP, blood creatinine	YES
Sun et al., 2012 (43)	60	30-60 mg/d	3	24h-UTP, blood creatinine	YES
Shen et al., 2011 (44)	30	60 mg/d	3	24h-UTP	YES
Hao et al., 2017 (45)	58	120 mg/d	3	24h-UTP, blood creatinine	YES
Li et al., 2018 (46)	62	1-1.5 mg/(kg·d)	3	24h-UTP, blood creatinine	No adverse event
Ma et al., 2020 (47)	102	60 mg/d	3	24h-UTP, blood creatinine	YES
Wang et al.2018 (22)	40	60 mg/d	6	24h-UTP, blood creatinine	YES
Kong et al., 2013 (48)	60	60 mg/d	6	24h-UTP, blood creatinine	YES
Lu et al., 2019 (49)	200	30-60 mg/d	6	Not reported	YES
Yu et al., 2011 (50)	129	60 mg/d	6	24h-UTP, blood creatinine	YES
Yang et al., 2013 (51)	60	1-1.5 mg/(kg·d)	6	Not reported	YES
Li et al., 2020 (52)	80	10-60 mg/d	6	24h-UTP, blood creatinine	YES
Zhou et al., 2019 (53)	200	30-60 mg/d	6	24h-UTP, blood creatinine	Not reported
Xu et al., 2017 (54)	72	10-60 mg/d	6	blood creatinine	YES
Gao et al., 2012 (55)	80	60 mg/d	6	24h-UTP, blood creatinine	YES
Chen et al., 2009 (56)	119	1-2 mg/(kg·d)	6	24h-UTP, blood creatinine	YES
Shan et al., 2013 (57)	70	1mg/(kg·d)	6	Not reported	YES
Zhou et al., 2019 (58)	122	10-60 mg/d	6	Not reported	YES

### Results With a Duration of 3-Month Treatment

#### Risk-of-Bias Assessment

Risk-of-bias assessment is shown in **Figure S1**. 24 h-UTP, blood creatinine, and AE outcome indicators were evaluated separately by the funnel plot, which revealed that no asymmetry existed in AEs, but significant publication bias was found for 24 h-UTP and blood creatinine (**Figures S2-4**).

#### 24 h-UTP

**Figure 2:**
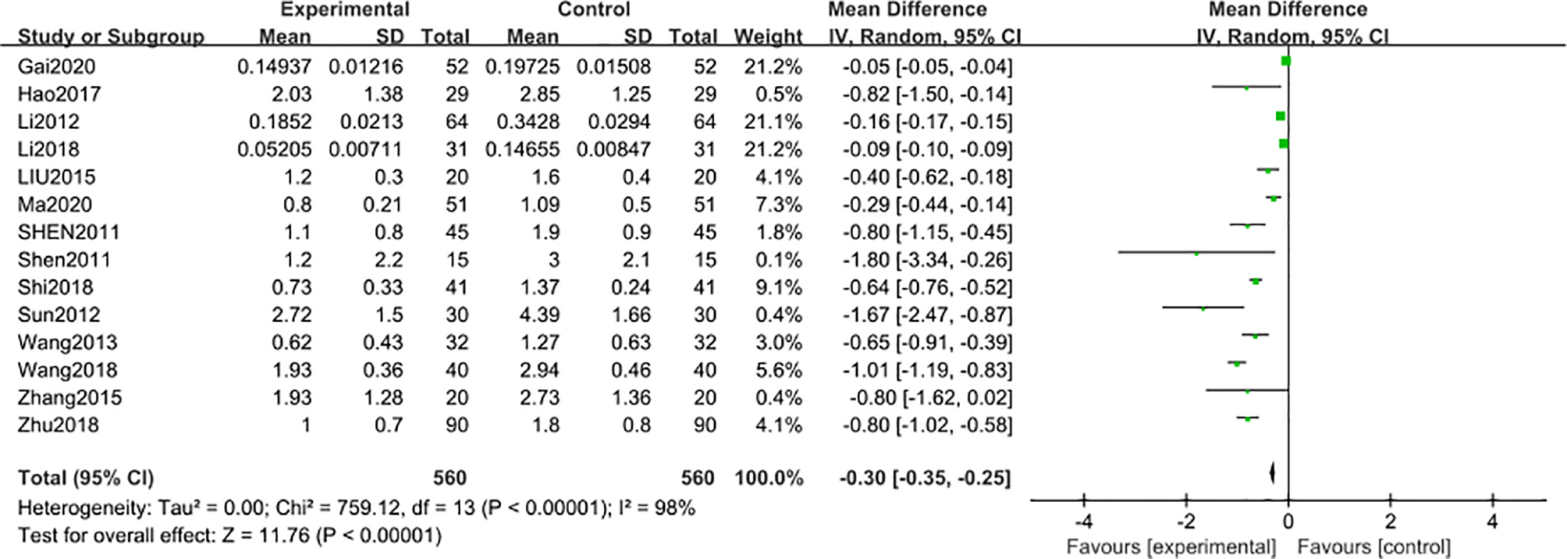
24 h-UTP after 3 months: comparison of treatment combined with TG against basic treatment.

Fourteen studies (29, 31–33, 38–47) including 1120 participants reported that the 24 h-UTP levels were significantly reduced [MD –0.30; 95% confidence interval (CI): –0.35 to –0.25; I^2^ = 98%] after 3-month of combined TG treatment compared with the non-TG regular treatment alone (**Figure 2**).

**Figure 3:**
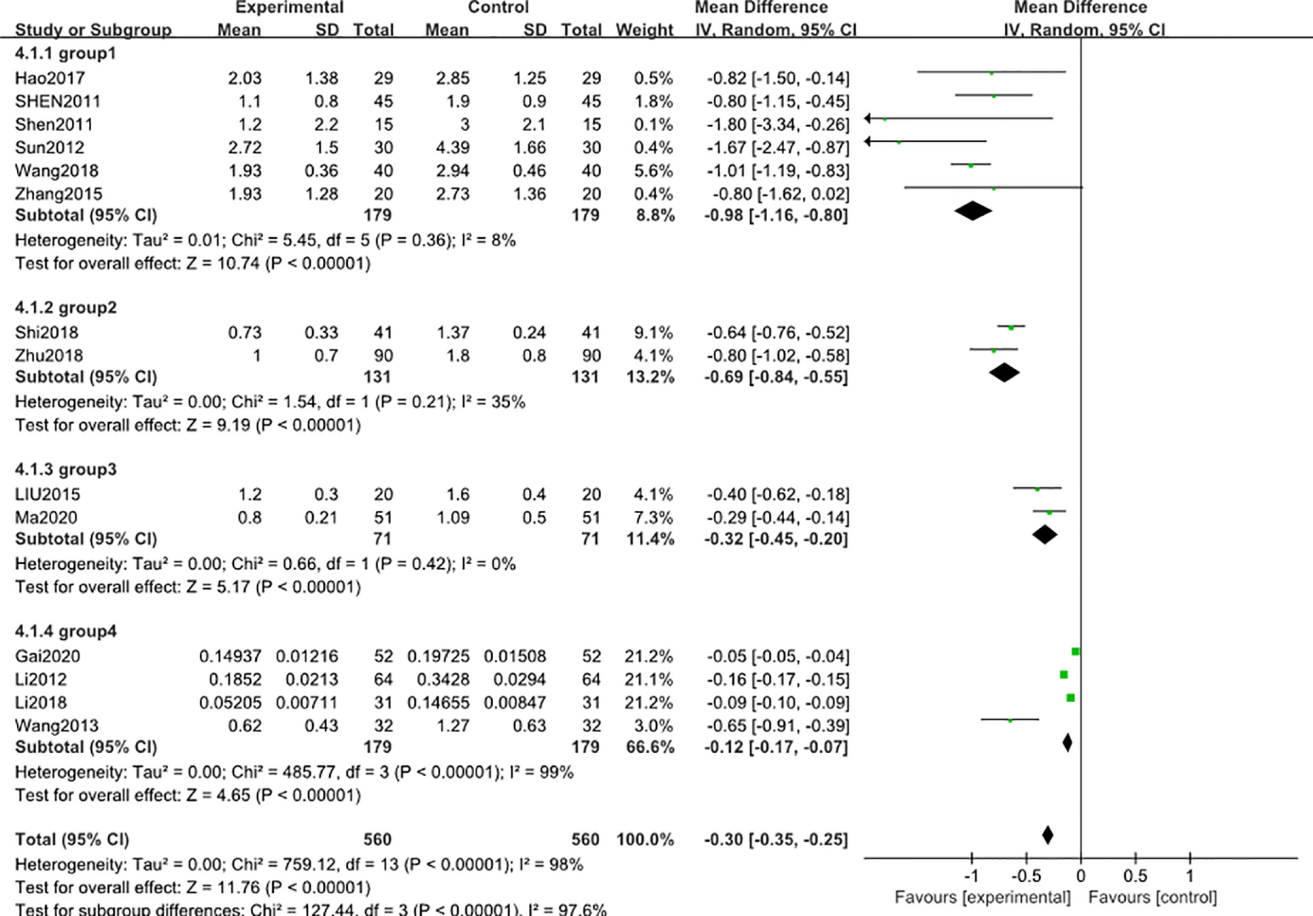
Subgroup analysis of 24 h-UTP level after 3 months: comparison of treatment combined with TG against basic treatment (group 1: range 3 g-, group 2: range 2.8-3 g, group 3: range 1.8-2.8 g, & group 4: range 0.2-1.5 g).

A subgroup analysis was conducted to reduce the heterogeneity in the results. The studies were divided into 4 subgroups (group 1: range >3 g, group 2: range 2.8-3 g, group 3: range 1.8-2.8 g, group 4: range 0.2-1.5 g) according to the baseline level of 24 h-UTP. The results showed that heterogeneity was reduced in the first three subgroups (**Figure 3**), suggesting that the difference in the 24 h-UTP baseline level was one of the critical heterogeneity sources. However, group 4 still had significant heterogeneity (I^2^ = 98%), which might be due to the limited number of available full-text literatures that could not be further categorized, resulting in the substantial variation at the baseline level of group 4 than other groups.

#### Blood Creatinine

**Figure 4:**
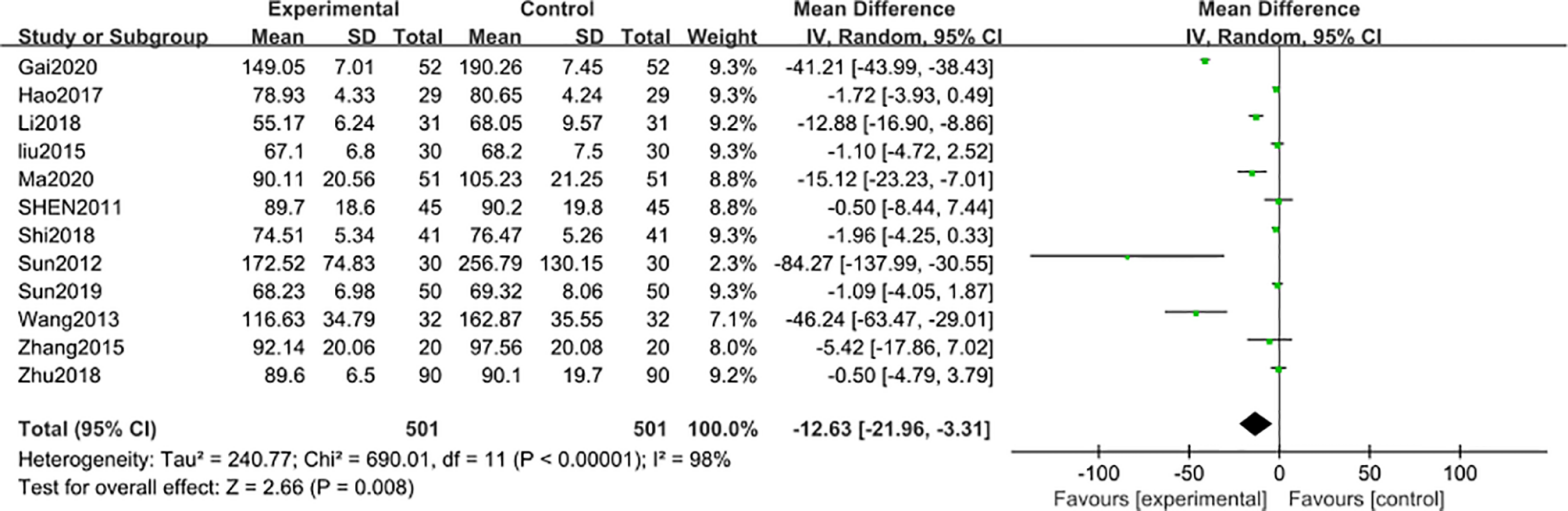
Blood creatinine level after 3 months: comparison of treatment combined with TG against basic treatment.

12 studies (31–33, 35, 37, 40–43, 45–47) including 1002 participants reported that the blood creatinine levels were significantly decreased [MD − 12.63; 95% CI: -21.96 to -3.31; I^2^ = 98%] after 3-month of combined TG treatment compared with the non-TG regular treatment alone. (**Figure 4**).

**Figure 5:**
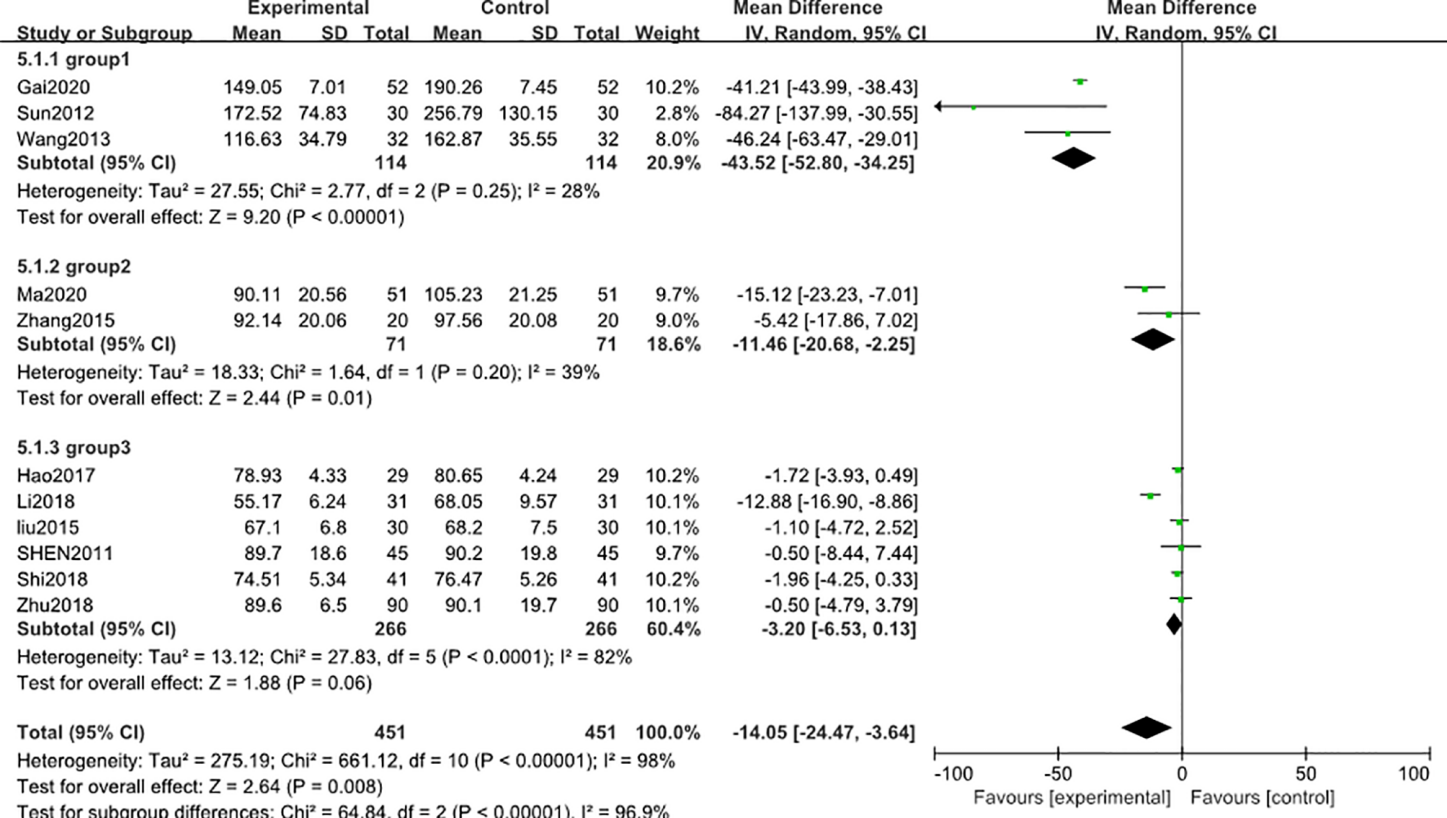
Subgroup analysis of blood creatinine level after 3 months: comparison of treatment combined with TG against basic treatment (group 1: range 160-200 μmol/L, group 2: range 99-130 μmol/L, group 3: range 60-92 μmol/L).

A similar subgroup analysis was conducted to reduce the heterogeneity in the results. Likewise, studies were divided into 3 subgroups according to the baseline level of blood creatinine (group 1: range 160-200 μmol/L, group 2: range 99-130μmol/L, group 3: range 60-92μmol/L). In the first two subgroups, the heterogeneity was reduced to an acceptable degree, but in the third subgroup, high heterogeneity was still existed (**Figure 5**). A *post hoc* sensitivity analysis was conducted to explore the heterogeneity origin in the third subgroup results. Notably, exclusion of a trial reported by Li (2018) (46)reduced the heterogeneity to a comparable level with respect to other subgroups [MD in blood creatinine -1.56; 95% CI: -2.92 to –0.20; I^2^ = 0%], suggesting that the difference in the blood creatinine baseline level of the patients was the main source of heterogeneity in this meta-analysis.

#### Adverse Reactions

**Figure 6:**
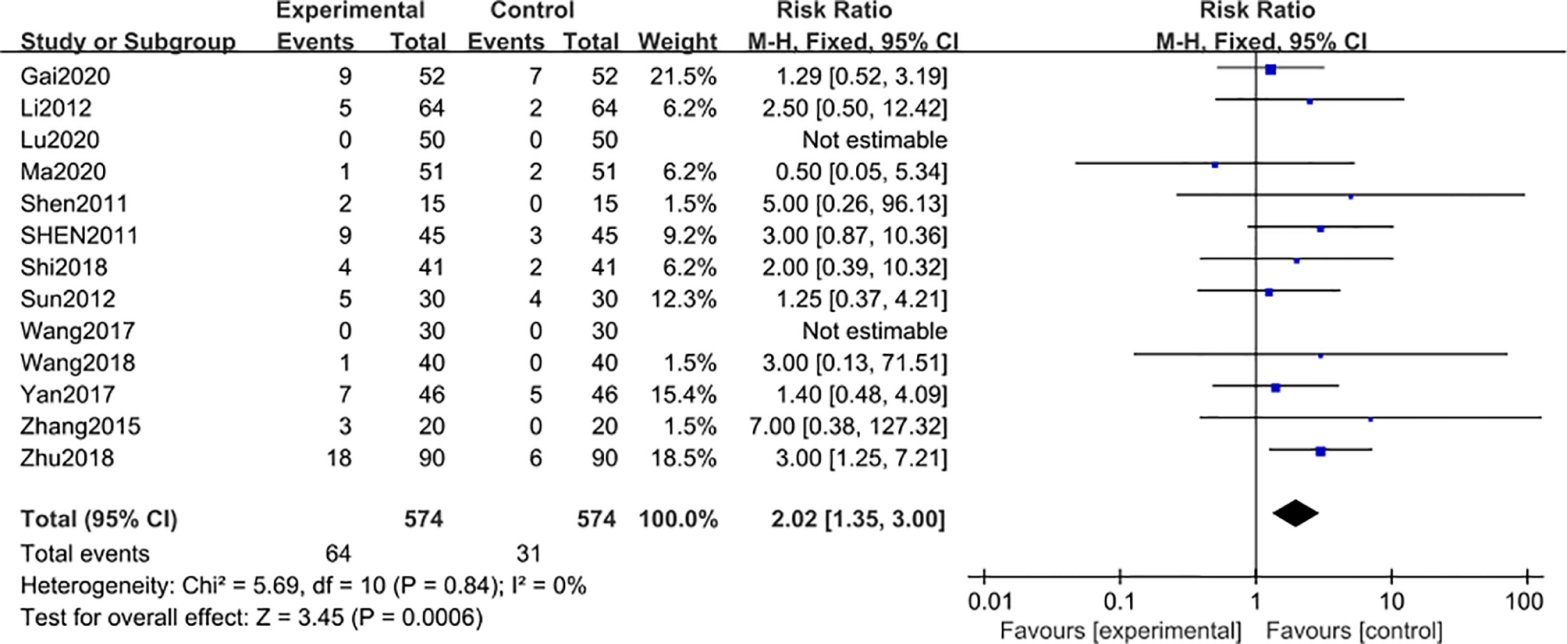
Adverse reaction events after 3 months: comparison of treatment combined with TG against basic treatment.

Adverse reaction events were reported in 13 studies (29, 30, 32–34, 36, 38, 40–44, 47), including 1148 subjects after 3-month of combined treatment with TG, which significantly increased the adverse reaction events [MD 2.02; 95% CI: 1.35 to 3.00; I^2^ = 0%], compared with non-TG regular treatments (**Figure 6**). This data was inconsistent with the findings of the included clinical studies involving TG administration that TG could not induce significant AEs even after 3 months of continuous treatment duration.

In addition, the AEs related to the treatments combined with TG mainly reflected symptoms of leukopenia and abnormal liver functions (**Table 2**).

**Table 2:** Statistics of adverse reaction events after 3-month treatment.

adverse reactionevents	treatment combined with TGtotal (574 patients)	basic treatmenttotal (574 patients)
gastrointestinal reactions	29(5.1%)	25(4.4%)
leukopenia	17(3.0%)^*^	0(0.0%)
abnormal liver function	14(2.4%)^*^	0(0.0%)
hypotension	2(0.3%)	4(0.7%)
hyperkalemia	1(0.2%)	0(0.0%)
fatigue	1(0.2%)	0(0.0%)
elevated creatinine	0(0.0%)	1(0.2%)
dizziness	0(0.0%)	1(0.2%)
**total**	64(11.2%)	31(5.5%)

*p < 0.01 (compared with basic treatment).

### Results With a Duration of 6-Month Treatment

#### Risk-of-Bias Assessment

The risk-of-bias assessment is shown in **Figure S5**. The adverse reaction outcome indicators were evaluated by the funnel plot, which showed no asymmetry (**Figure S6**).

#### 24 h-UTP

**Figure 7:**
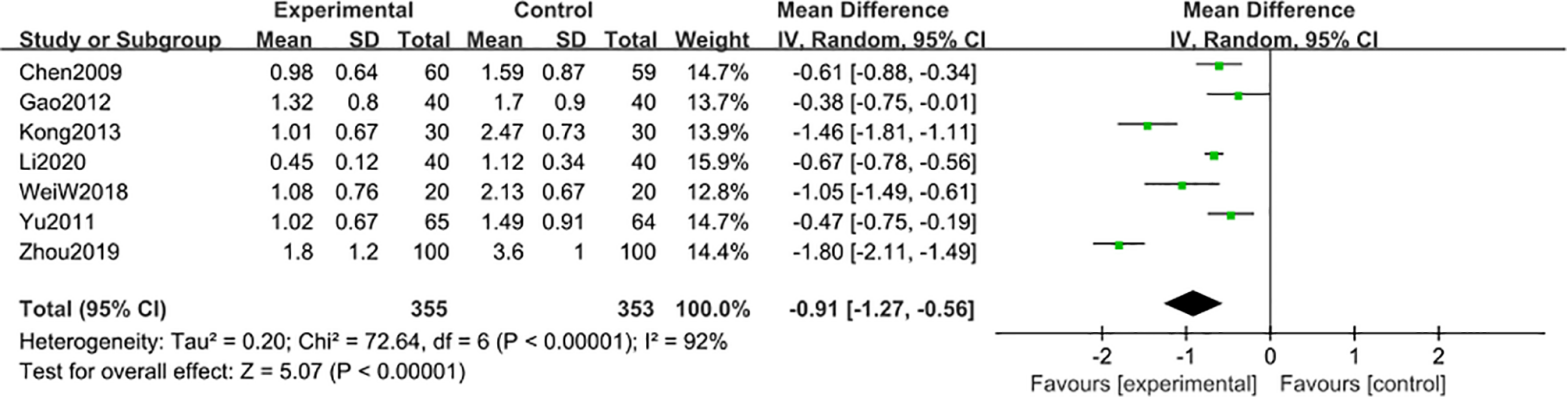
24 h-UTP level after 6 months: comparison of treatment combined with TG against basic treatment.

7 studies (22, 48, 50, 52, 55, 56, 58) including 708 participants reported that the 24 h-UTP after 6-month of treatment combined with TG was significantly reduced [MD -0.91; 95% CI: -1.27 to -0.56; I^2^ = 92%] (**Figure 7**).

**Figure 8:**
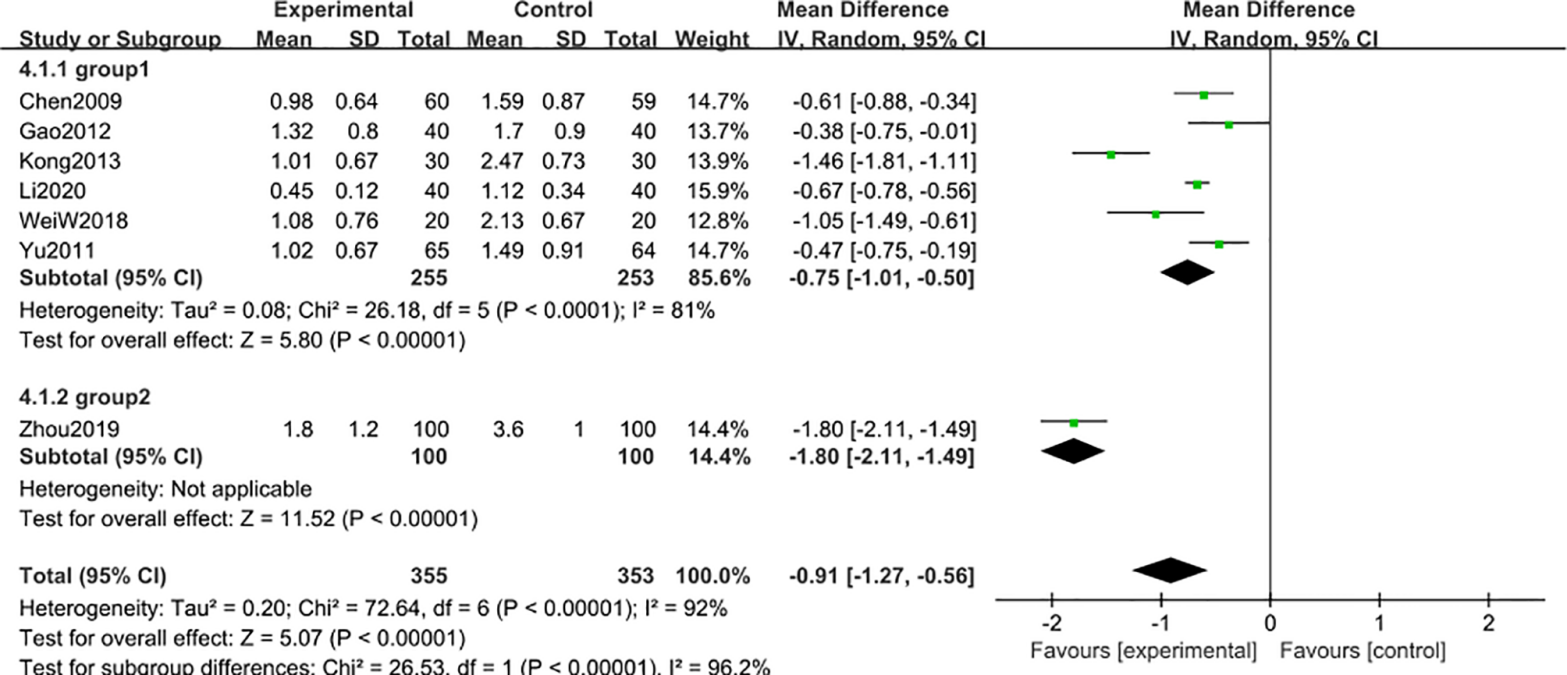
Subgroup analysis of 24 h-UTP level after 6 months: comparison of treatment combined with TG against basic treatment (group 1: range 2-3 g, group 2: range 4-5 g).

A subgroup analysis was conducted to reduce obvious heterogeneity. The studies were divided into 2 subgroups (group 1: range 2-3 g, group 2: range 4-5 g), according to the baseline level of 24 h-UTP. However, subgroup 1 still exhibited significant heterogeneity (I^2^ = 81%), while subgroup 2 involved only one article, thus could not be analyzed for heterogeneity. To explore the heterogeneity in subgroup 1, a *post hoc* sensitivity analysis was conducted, resulting in the exclusion of a trial reported by Kong et al. (48), thereby reducing the heterogeneity [MD -0.62; 95% CI: -0.77 to –0.47; I^2^ = 42%] (**Figure 8**), suggesting that the difference in the 24 h-UTP baseline was the main source of heterogeneity, as was observed for 3-month treatment duration.

#### Blood Creatinine

**Figure 9:**
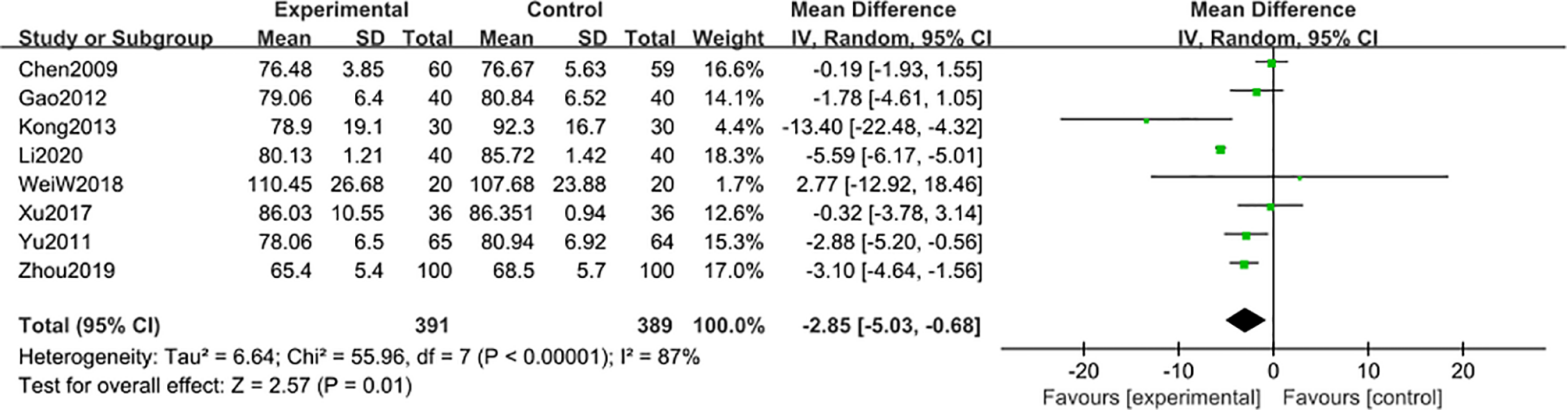
Blood creatinine level after 6 months: comparison of treatment combined with TG against basic treatment.

**Figure 10:**
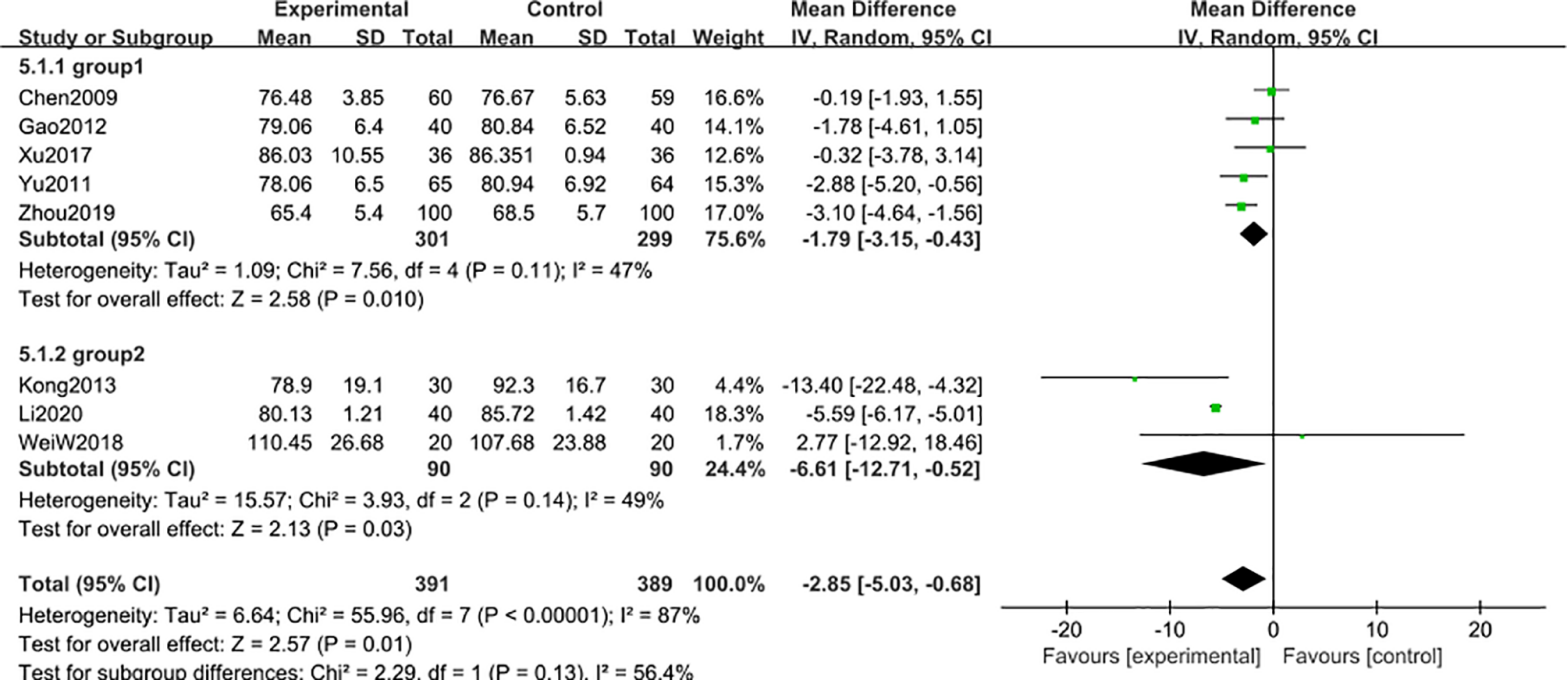
Subgroup analysis of blood creatinine after 6 months: comparison of treatment combined with TG against basic treatment (group 1: range 70-88 μmol/L, group 2: range 94-109 μmol/L).

8 studies (47, 48, 50, 52, 54–56, 58) including 780 participants reported that blood creatinine level was reduced after 6-month of treatment combined with TG [MD -2.85; 95% CI: -5.03 to -0.68%; I^2^ = 87%], compared with the non-TG regular treatment (**Figure 9**). Similar subgroup analysis was used to reduce the heterogeneity to an acceptable degree after dividing the articles into two groups (group 1: range 70-88 μmol/L, group 2: range 94-109 μmol/L) (**Figure 10**), and the results showed that the heterogeneity was mainly sourced from variation at a baseline level of blood creatinine.

#### Adverse Reactions

**Figure 11:**
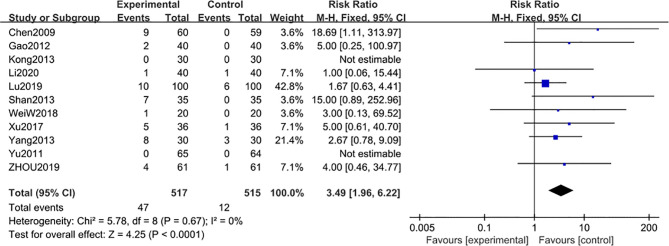
Adverse reaction events after 6 months: comparison of treatment combined with TG against basic treatment.

Eleven studies (22, 48–52, 54–58) including 1032 participants, reported the adverse reaction events after 6-month of treatment combined with TG. Compared with the regular treatment, TG treatment could significantly increase the adverse reaction events [MD 3.49; 95% CI: 1.96 to 6.22; I^2^ = 0%] (**Figure 11**). The results revealed that AEs after 6 months of TG treatment were mainly manifested as symptoms of leukopenia and abnormal liver functions, further confirming the results from the previous studies that long-term use of TG could lead to liver injury and leukopenia (**Table 3**).

**Table 3:** Statistics of adverse reaction events after 6-month treatment.

adverse reactionevents	treatment combined with TGtotal (517 patients)	basic treatmenttotal (515 patients)
gastrointestinal reactions	4(0.8%)	2(0.4%)
leukopenia	14(2.7%)^*^	0(0.0%)
abnormal liver enzymes	25(4.8%)^*^	0(0.0%)
hypoglycemia	1(0.2%)	1(0.2%)
elevated creatinine	0(0.0%)	3(0.6%)
hyperkalemia	0(0.0%)	6(1.2%)
menstrual disorders	3(0.6%)	0(0.0%)
**total**	47(9.1%)	12(2.3%)

*p < 0.01 (compared with basic treatment).

### GRADE Assessment

According to the Grading of Recommendations Assessment, Development, and Evaluation (GRADE), the quality of evidence for AE was moderate, while for 24 h-UTP and blood creatinine indicators were low (**Table 4**). Although the literature quality was not sufficiently high in this meta-analysis, however, a great number of participants were included in this study. This meta-analysis and systematic literature review, therefore, suggest that long-term application of TG could reduce the 24 h-UTP and blood creatinine level of patients with DN to normal levels, but at the same time, it can also induce considerable AEs, further complicating DN pathogenesis.

**Table 4:** GRADE assessment of quality of evidence for outcomes.

Duration(months)	Outcomes	Participants	studies	Risk of bias	Inconsistency	Indirectness	Imprecision	Publication bias	Overall quality of evidence
3	24h-UTP	1120	14	Serious^1^	No serious	NO serious	NO serious	Serious^2^	LOW^1,2^, due to risk of bias, publication bias
3	blood creatinine	1002	12	Serious^1^	No serious	NO serious	NO serious	Serious^2^	LOW^1,2^, due to risk of bias, publication bias
3	AR	1148	13	Serious^1^	No serious	NO serious	NO serious	NO serious	MODERATE^1^, due to risk of bias
6	24h-UTP	708	7	Serious^1^	No serious	NO serious	NO serious	Serious^2^	LOW^1,2^, due to risk of bias, publication bias
6	blood creatinine	780	8	Serious^1^	No serious	NO serious	NO serious	Serious^2^	LOW^1,2^, due to risk of bias, publication bias
6	AR	1032	11	Serious^1^	No serious	NO serious	NO serious	NO serious	MODERATE^1^, due to risk of bias

1.The random and blind methods were of poor quality. 2.There was publication bias.

## Discussion

*Supplement to Compendium of Materia Medica*, an ancient traditional Chinese medical book authored by Zhao Xuemin (Qing dynasty) has documented that TW can be used in the treatment of tympanites, edema, epigastric fullness, jaundice, malaria that persists for a long time and also in traumatic injury (59), indicating that TW has been used as a critical traditional medicine in treating several fatal diseases for centuries. Notably, the possible toxicities of TW administration were also recorded at the beginning of its clinical applications (60).

In recent years, the active ingredient of TW extract, TG, has been purified and subsequently tested for its toxicity. Emerging studies have shown that TG could significantly lower the levels of 24 h-UTP and blood creatinine even in patients with the high initial baseline values, while the degree of reduction was smaller in patients with the lower initial baseline values, suggesting that TG may have a better efficacy for severe DN patients. Thus, though toxic, TG has still been used in clinical practice.

Studies on the long-term treatment effect show that after applying TG for DN 12 months, the 24 h-UTP of the patients were still significantly decreased (61). But the amount of relevant literature is insufficient to be included in this system reviews.

To maintain the balance between efficacy and safety profiles of TG usage, special attention should be paid to the appropriate dosage and duration of treatment course precisely depending on the pathological symptoms of individual patients. Unfortunately, comprehensive and high-quality studies are still lacking for accurate clinical application of this important drug leading to controversial medicine guidance, clinical practice and random occurrences of adverse reactions. Although the medicine guidance recommends the course of treatment with TG should not be more than 3 continuous months, however, in several clinical investigations, patients have reportedly undertaken TG treatment for up to 6 continuous months.

Our results show that though AE profiles were very similar between 3-month and 6-month of course duration, the occurrences of severe AEs were relatively much higher after 6 months. Moreover, even after 3 months of TG treatment, severe AEs can happen at a rate as high as 5.4% of total patients, suggesting that better safety can be achieved by reducing the course duration even less than 3 continuous months. To our regret, there are not enough eligible research studies available on AE occurrence in relation to the duration of treatment with TG to allow us to comprehensively investigate the cause-effect relationship. Despite this, our work could still reveal that the published guidelines on the course of treatment with TG from both medicine guidance and clinical practice should be ameliorated, and more attention should be paid to the severe AEs related to TG medication, and symptomatic treatment should be applied immediately at the onset of these severe AE symptoms.

TW contains over 400 active ingredients. As an active ingredient of TW extract, TG’s combination is simplified to a series of glycosides, which decrease the toxicity, but still lack of accurate pharmacological properties and manufactural quality. At present, the standard and proportion of TG in the market are lack of consistency, so the chemical composition produced by different manufacturers may be different (62). This may also contribute to the adverse reactions of TG.

In the absence of high-quality evidence for TG-associated adverse reactions, theories of traditional Chinese medicine practice stating that ‘Stabilize that condition without excessive medical treatment’ could be employed to adjust the appropriate dosage of TG, while the combination with herbal extracts containing leukocyte proliferation agents, e.g., *Cordyceps sinensis* (63), *Ganoderma lucidum* (64) and liver-protecting medicine, e.g., Milk thistle (65), *Polyporus umbellatus* (66) extracts may be an alternative therapeutic approach to alleviate AEs and improve the safety profile of TG application.

Taken together, this meta-analysis and systematic review suggest that more comprehensive and high-quality clinical investigations are urgently warranted to establish the treatment guidelines for TG and its related adverse reactions with respect to the patient’s clinical stage of DN progression as well as comorbid symptoms to broaden the therapeutic application of this important natural medicine.

To further investigate the balance of efficacy and adverse effects, firstly, we would consider analyzing the differences among more course subgroups of DN patients and the relationships among clinical efficacy, AEs, and individual difference. Researchers should regulate the dosage, identify the manufacturers, and focus on the relationship between the duration treatment and AEs. Secondly, more clinical endpoints should be considered to evaluate the efficacy of TG, like Glomerular filtration rate (GFR).

From a real-world research perspective, investigators also need pay attention to the standard of randomization methods, blinding, and allocation concealment to standardize RCTs. At the same time, negative results should be properly reported to avoid publication bias. Further controlled studies should be done on the age, stage, course of DN patients to evaluate what difference is made in the efficacy of TG for different populations.

## Limitations

This study also suffers from certain inevitable limitations that require further consideration. Firstly, and most importantly, publications on the 24 h-UTP and blood creatinine indices after 3-month course of treatment with TG are significantly biased. And secondly, high-quality research studies relating to TG dosage and induced adverse reactions are not enough to firmly conclude on specific AEs due to particular TG treatment course.

Furthermore, most of the systematic reviews focused on efficacy or effectiveness. The methodology for conducting systematic reviews of beneficial effects from RCTs is well established, whereas the methods for systematically reviewing randomized or observational data on AEs are less well developed and less often used (67). Thus, researchers who conduct systematic reviews have limited sources of guidance, such as the suggestions offered by the Cochrane Collaboration. Moreover, the pre-determined harmful effects of interest were known to be under-reported in RCTs (68). These questions lead us to some innate limitations in this systematic review.

Although some researchers are accustomed to using 24 h-UTP and blood creatinine as the main surrogate biomarkers to evaluate the prognosis of renal disease in RCTs, 24 h-UTP and blood creatinine still have great limitations as clinical endpoint.

Because of the kidney’s ability of compensate, when patients with renal impairment, the blood creatinine may still be in a normal level. Blood creatinine and 24 h-UTP does not reflect the long-time state of renal function well, so risks would be produced by using them to determine the efficacy. GFR is a better indicator for evaluating renal function. But, GFR is rarely used in clinical studies to evaluate the efficacy of TG. Recently, a growing number of studies have shown that the sensitivity of cystatin C to the decrease of GFR is better than that of blood creatinine, especially in the early stage of renal injury (69–71). Unfortunately, cystatin C is not widely applied at present, and relevant literature remain scarce. Our understanding of DN may greatly benefit from more detailed investigation into these surrogate indicators.

## Conclusion

Our results have revealed that symptomatic treatments combined with TG can significantly lower 24 h-UTP and blood creatinine levels in DN patients than the basic treatment without TG can do, confirming the efficacy of TG. While forest plots of these two indicators have exhibited that apparent heterogeneity remains even after subgroup and sensitivity analyses, however, there are ways to reduce the heterogeneity to an acceptable degree.

Regarding the induction of adverse side-effects, patients from both 3-month and 6-month groups undertaking TG medications showed critical AE onsets, e.g. leukopenia and abnormal liver functions, especially more aggressively in patients of the 6-month treatment group. However, these results were inconsistent with the published reports we included in this study, indicating no significant differences in AE profiles between the experimental group and placebo-treated or control group. According to the GRADE assessments, the quality of evidence from these articles was low, primarily might be due to the insufficient sample size and error-prone experimental designs. Thus, the descriptions of AEs from the medicine guidance should be really concerning in clinical practice.

Importantly, the occurrence of AEs was very similar after 3-month (64/574, 11.1%) and 6-month (47/517, 9.1%) of TG treatment durations. However, the incidence of severe AEs after 6-month treatment with TG (39/517, 7.5%) reportedly had 39% increment than that happened after 3-month treatment with TG (31/574, 5.4%). The total percent of AEs in treatment with TG didn’t greatly increase with TG, but as the course of treatment lasted, severe AEs were more likely to happen.

In summary, our work showed that TG was therapeutically effective in the treatment of DN-related symptoms like proteinuria, high serum creatinine, but insufficient sample sizes and inappropriate experimental designs caused non-significant AE differences between the experimental and control groups in several studies. AE occurrence rate was found nearly constant as the medicine duration increased, however, the percent of severe AEs after 6 months of treatment was 1.39 times more than that after 3 months.

## Data Availability Statement

The original contributions presented in the study are included in the article/**Supplementary Material**. Further inquiries can be directed to the corresponding authors.

## Author Contributions

YLi and RM wrote the manuscript. YLi and RM selected the trials. ZD and JZ extracted the data. YZ and LZ assessed the quality of the studies. ZH and YZ assessed the quality of the evidence. YLiu, PM, and YLi performed the statistical analysis. DH and YX conceived of the study, and all other authors critically reviewed the report. All authors contributed to the article and approved the submitted version.

## Funding

National Key R&D Program of China, No. 2019YFC1709801.

## Conflict of Interest

The authors declare that the research was conducted in the absence of any commercial or financial relationships that could be construed as a potential conflict of interest
