# Supplementary material for: Efficacy and Safety of Tripterygium Glycoside in the Treatment of Diabetic Nephropathy: A Systematic Review and Meta-Analysis Based on the Duration of Medication
Source: Front Endocrinol (Lausanne). 2021 Apr 20;12:656621. doi: 10.3389/fendo.2021.656621 (PMC8095376; doi:10.3389/fendo.2021.656621)
Supplement: Supplementary file 1 [file DataSheet_1.docx]

**电子检索：**

**1. 主题词的同义词扩展：**

使用维普、PUBMED对主题词进行扩展

**1.1 维普对雷公藤多苷片和糖尿病肾病、糖尿病肾脏病同义词扩展**

**1.1.1雷公藤多苷片同义词：**雷公藤多苷片

**1.1.2糖尿病肾病、糖尿病肾脏病同义词：**糖尿病肾病、糖尿病性肾病、糖尿病性肾小球硬化症、糖尿病肾脏疾病、糖尿病肾脏病变、糖尿病性肾脏疾病、糖尿病肾脏病

**1.2 PUBMED对“tripterygium glycosides”和“diabetic kidney disease”****“diabetic nephropathy”同义词扩展**

**1.2.1 tripterygium glycosides同义词：**tripterygium glycosides

**1.2.2 diabetic kidney disease、“diabetic nephropathy” 同义词:** diabetic nephropathy、diabetic kidney disease

**2.检索数据库**

**数据库：**知网、万方、维普、SinoMed、PUBMED、COCHRANE LIBRARY、中国临床试验注册中心、WHO国际临床试验注册平台

**3.检索日期：**截止2020年11月05日

**4.检索结果**

**4.1知网**

SU=(雷公藤多苷片) AND SU=(糖尿病肾病+糖尿病性肾病+糖尿病性肾小球硬化症+糖尿病肾脏疾病+糖尿病肾脏病变+糖尿病性肾脏疾病+糖尿病肾脏病) AND FT=(随机)69篇

**4.2万方**

主题:（雷公藤多苷片）*主题:(糖尿病肾病 or 糖尿病性肾病 or 糖尿病性肾小球硬化症or糖尿病肾脏疾病or糖尿病肾脏病变or糖尿病性肾脏疾病or糖尿病肾脏病) *(随机)88篇

**4.3 维普**

(M=雷公藤多苷片)*(M=糖尿病肾病+M=糖尿病性肾病+M=糖尿病性肾小球硬化症+M=糖尿病肾脏疾病+M=糖尿病肾脏病变+M=糖尿病性肾脏疾病+M=糖尿病肾脏病)*(R=随机)53篇

**4.4 SinoMed**

(雷公藤多苷片) AND (糖尿病肾病 OR 糖尿病性肾病 OR 糖尿病性肾小球硬化症 OR 糖尿病肾脏疾病 OR 糖尿病肾脏病变 OR 糖尿病性肾脏疾病 OR 糖尿病肾脏病) AND (随机)69篇

**4.5 PUBMED**

(((diabetic nephropathy) OR (diabetic kidney disease)) AND (tripterygium glycosides)) AND (random)10篇

**4.6 COCHRANE LIBRARY**

(tripterygium glycosides):ti,ab,kw AND (diabetic nephropathy OR diabetic kidney disease):ti,ab,kw AND (random):ti,ab,kw 3篇

**4.7中国临床试验注册中心(http://www.chictr.org.cn)**

以“雷公藤多苷片”为检索词，共搜到3项关于雷公藤多苷片的临床试验，3项临床试验均与雷公藤多苷片治疗糖尿病肾病无关，3项

**4.8 WHO国际临床试验注册平台（https://www.who.int/ictrp/en/）**

以“tripterygium glycosides” 为检索词，共搜到8项关于雷公藤多苷片的临床试验，其中1项试验与雷公藤多苷片治疗糖尿病肾病有关，但无实验结果。8项
