# Supplementary material for: Efficacy and Safety of Tripterygium Glycoside in the Treatment of Diabetic Nephropathy: A Systematic Review and Meta-Analysis Based on the Duration of Medication
Source: Front Endocrinol (Lausanne). 2021 Apr 20;12:656621. doi: 10.3389/fendo.2021.656621 (PMC8095376; doi:10.3389/fendo.2021.656621)
Supplement: Supplementary file 2 [file DataSheet_2.doc]

# Supporting Information

### Table S1. General characteristics of literature screening

| **characteristics** | **Details** |
| --- | --- |
| **Inclusion criteria** | ① The diagnostic criteria of DN were in accordance with 2007 National Kidney Foundation Kidney of Disease Outcomes Quality Initiative (NKF-K/DOQI)  ② The intervention in the treatment group: TG were used on the basis of the control group; the control group: basic treatment  ③ The research literature reported at least one of the following outcomes: 24h-UTP, blood creatinine, adverse reactions  ④ The research type was randomized controlled trail (RCT)  ⑤ The treatment lasted for 3 or 6 months |
| **Exclusion criteria** | ① Patients with no diabetic nephropathy in studies  ② The interventions in the treatment group or control group did not accord with the inclusion criteria  ③ Not reported outcomes mentioned of the inclusion criteria  ④ Non-RCT  ⑤ repetitive study |


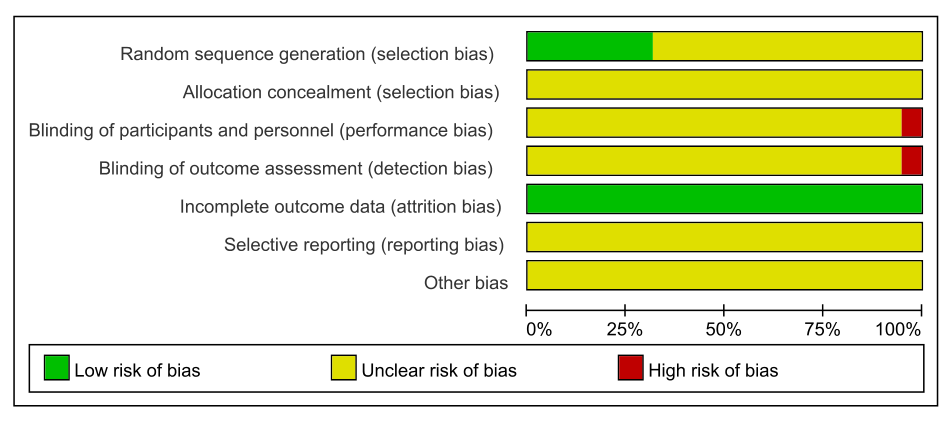


### Figure S1. Risk of bias across studies after 3 months.


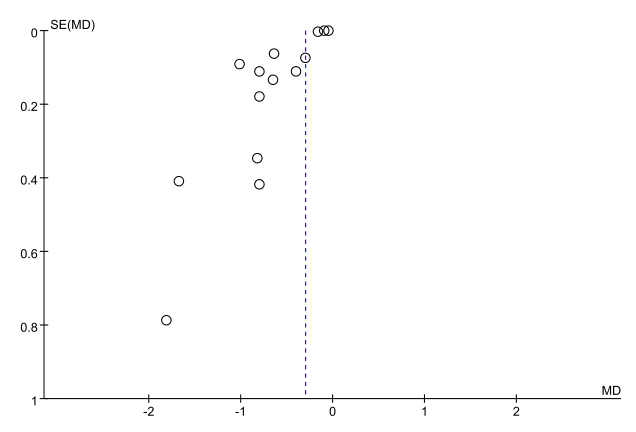


### Figure S2. 24h-UTP: funnel plot of 3 months.


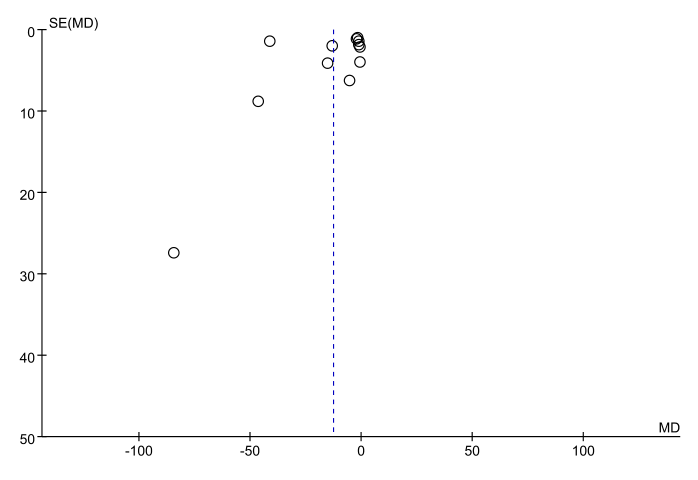


### Figure S3. Blood creatinine: funnel plot of 3 months.


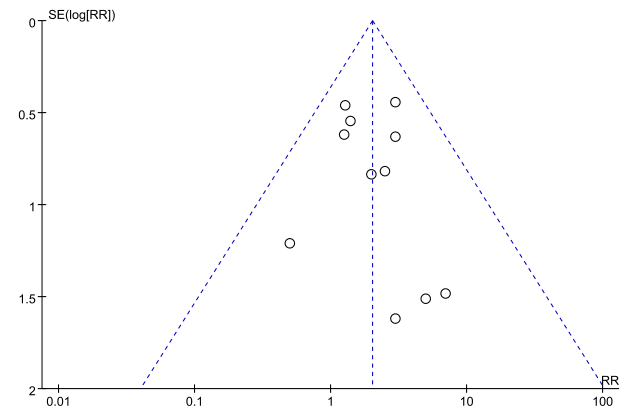


### Figure S4. Adverse reaction: funnel plot of 3 months.


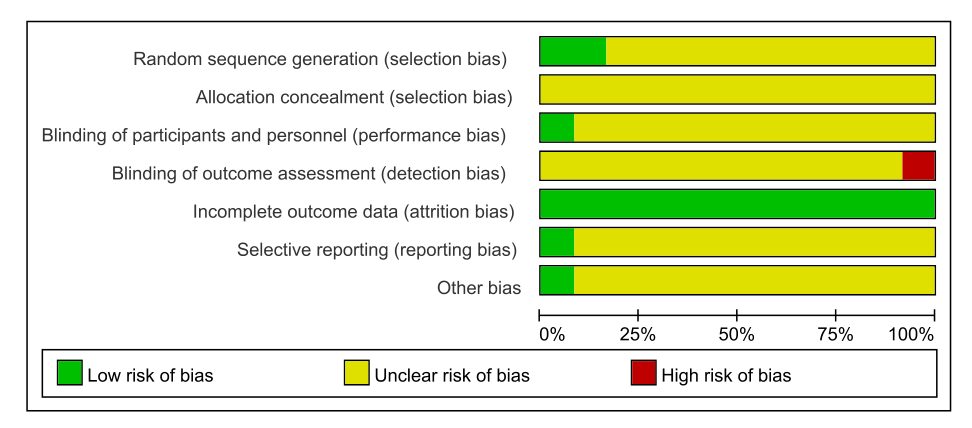


### Figure S5. Risk of bias across studies after 6 months.


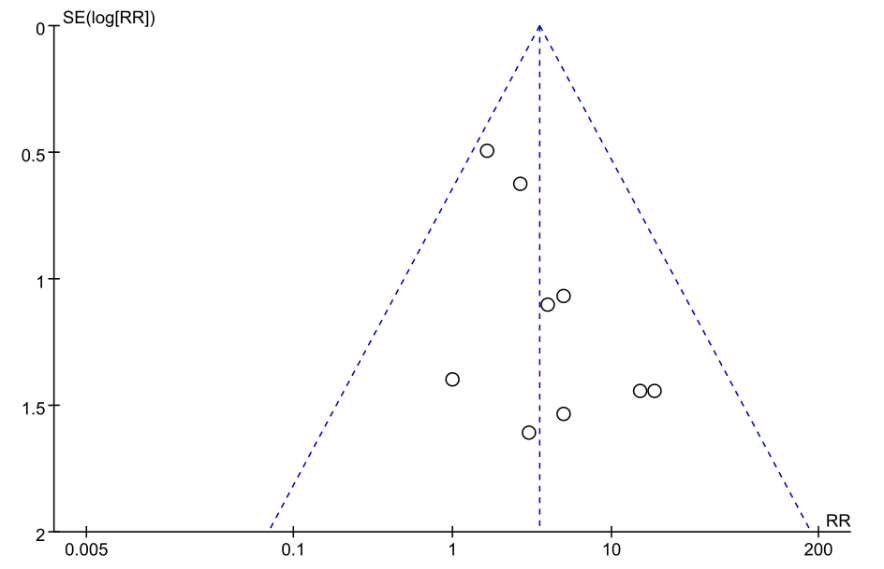


### Figure S6. Funnel plot of adverse reaction events after 6 months.
